# Identification of novel antioxidant gene signature to predict the prognosis of patients with gastric cancer

**DOI:** 10.1186/s12957-021-02328-w

**Published:** 2021-07-20

**Authors:** Jianhua Wu, Xuan Wang, Nan Wang, Li Ma, Xin Xie, Hao Zhang, Huafeng Kang, Zhangjian Zhou

**Affiliations:** 1 Department of Oncology, The Second Affiliated Hospital of Xi’an Jiaotong University, Xi’an, China; 2 Department of Surgical Oncology, The First Affiliated Hospital of Xi’an Jiaotong University, Xi’an, China

**Keywords:** Antioxidants, Gastric cancer, Gene signature, Prognosis, Nomogram model

## Abstract

**Background:**

Gastric cancer (GC) commonly relates to dismal prognosis and lacks efficient biomarkers. This study aimed to establish an antioxidant-related gene signature and a comprehensive nomogram to explore novel biomarkers and predict GC prognosis.

**Methods:**

Clinical and expression data of GC patients were extracted from The Cancer Genome Atlas database. Univariate and multivariate Cox analyses were utilized to construct a score-based gene signature and survival analyses were conducted between high- and low-risk groups. Furthermore, we established a prognostic nomogram integrating clinical variables and antioxidant-related gene signature. Its predictive ability was validated by Harrell' concordance index and calibration curves and an independent internal cohort verified the consistency of the antioxidant gene signature-based nomogram.

**Results:**

Four antioxidant-related genes (CHAC1, GGT5, GPX8, and PXDN) were significantly associated with overall survival of GC patients but only two genes, CHAC1 (HR = 0.803, *P* < 0.05) and GPX8 (HR = 1.358, *P* < 0.05), were confirmed as independent factors. A score-based signature was constructed and could act as an independent prognosis predictor (*P* < 0.05). Patients with lower scores showed significantly better prognosis (*P* < 0.05). Comprehensive nomogram combining the antioxidant-related gene signature and clinical parameters (age, gender, grade, and stage) was established and effectively predicted overall survival of GC patients [3-year survival AUC = 0.680, C index = 0.665 (95% CI 0.614–0.716)]. The independent internal validation cohort verified the reliability and good consistency of the model [3-year survival AUC = 0.703, C index = 0.706 (95% CI 0.612–0.800)].

**Conclusions:**

Innovative antioxidant-related gene signature and nomogram performed well in assessing GC prognoses. This study enlightened further investigation of antioxidant system and provided novel tools for GC patient management.

**Supplementary Information:**

The online version contains supplementary material available at 10.1186/s12957-021-02328-w.

## Background

In recent years, gastric cancer (GC) remains a common cancer worldwide. There are around 27,600 newly diagnosed GC patients and 11,010 GC related deaths in the USA in 2020 [1]. Although recommended life style and combined treatment have helped improve the clinical outcome of GC patients, general 5-year overall survival remains approximately 20% globally [2]. This poor clinical outcome of GC patients is mainly due to the diagnosis at late stages [3]. Therefore, it is urgently needed to find promising biomarkers for screening patients at high risk and build a risk model to evaluate their prognosis to guide clinical practice.

There have been researches exploring biomarkers including gene expression profiles emphasized in GC prognosis, most of which demonstrated that the differentially expressed genes were associated with patients overall survival [4, 5]. In addition, more and more studies have tried to establish molecular signatures or combine multiple biomarkers to present a more convincing prediction of GC prognosis [6, 7]. Besides, nomograms were developed incorporating these prognostic biomarkers and clinical variables to further improve prediction accuracy [8–10].

Researchers have noticed that reactive oxygen species (ROS) and antioxidants participate in carcinogenesis and cancer treatment [11]. Limited ROS can be anti-tumorigenic while excessive levels can be promotive [12]. Similarly, recent studies have found conflicting results about the role of antioxidants in cancer treatment [13, 14]. Therefore, more studies are needed to explore its functions. Meanwhile, clinical researchers have made use of antioxidants to develop new therapies for GC or to explain pharmacologic action [15, 16], and some of them further studied the expression profiles of the antioxidant-related genes in GC, which might affect the function of ROS and antioxidants [17, 18]. Antioxidant-related genes might be promising biomarker candidates and informative to prognostic prediction.

However, relevant studies on antioxidant-related gene signature are few and its prognostic significance in GC remains unexplored. Hence, in this study, based on the data from The Cancer Genome Atlas (TCGA) database, the predictive antioxidant-related genes were identified and a risk model was constructed to evaluate the outcome of GC patients, which also helps enlighten the potential mechanisms of molecular antioxidant in gastric cancer progression and offer more potential targets for the treatment. Furthermore, a comprehensive nomogram on the basis of the antioxidant-related gene signature and clinical variables was built to assess the prognoses of GC patients effectively in clinical practice.

## Methods

### Data collection

**Fig. 1:**
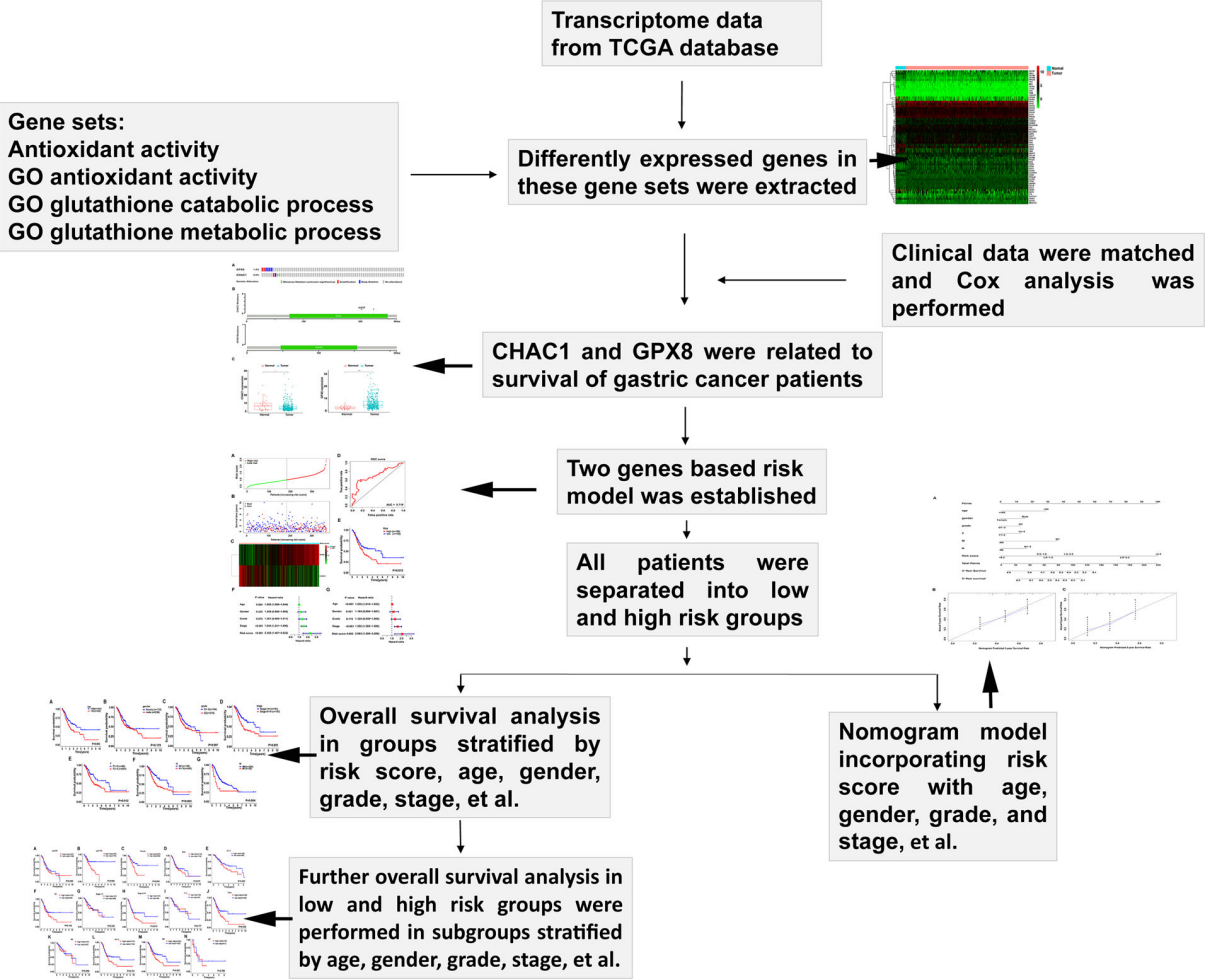
The flow chart and the main process of analysis in this study

Firstly, clinical information of GC patients and the gene expression data were extracted and matched from TCGA database (https://portal.gdc.cancer.gov/). A flow chart was drawn to show all the analysis procedure in this study (Fig. 1).

### Screening of the differentially expressed genes

From the website of gene set enrichment analysis (GSEA, https://www.gsea-msigdb.org/gsea/index.jsp), we obtained four antioxidant-related gene sets (antioxidant activity, GO antioxidant activity, GO glutathione catabolic process and GO glutathione metabolic process). Then, under the R environment, gene expression data from TCGA database were screened and proceeded with “limma” R package to select the differently expressed antioxidant-related genes in GC patients [19].

### Establishment of the gene signature

With “survival” R package, univariate and multivariate Cox regression analyses were performed to select the genes with independent prognostic value and a linear risk score formula was established. Risk scores of all the GC samples can be calculated as follows: risk parameter = ∑ (expression of gene n × βn) (n represents the number of independent prognostic genes and β represents regression coefficients). All the GC patients were assigned risk scores and by group median risk score, they were subsequently divided into high- or low-risk teams. Log-rank tests and Kaplan-Meier curves of the two groups validated the prognostic significance of the risk score. Furthermore, we conducted overall survival analyses in stratified subgroups to further explore the prognostic ability of risk score by “survival” and “survminer” R package.

### Construction and evaluation of the nomogram

A comprehensive nomogram predicting survival probability of GC patients was built by integrating antioxidant-related gene signature and clinicopathologic variables, which was conducted by “rms” R package. Based on regression analyses, the nomogram can predict the 3- and 5-year survival probability of GC patients. To assess its performance, Harrell’ C-index, AUC of ROC, and calibration curves were generated. Harrell’ C-index is positively related to the accuracy of nomogram and an ideal calibration graph should be close to 45-degree dotted line. Besides, an internal validation from TCGA database was performed to further confirm the feasibility. Bootstrap resampling was used in these activities.

### Statistical analysis

Cox analyses aimed to select the variables with independent prognostic value and Kaplan-Meier curve analysis was performed to evaluate clinical significance of risk factors. Based on R software version 4.0.2 (http://www.R-project.org/) and Excel software (Microsoft Corporation, California), statistical analyses were properly conducted by flexible statistical methods. R packages “limma,” “survival,” “rms,” and “survminer” were utilized for organizing data, Cox analyses, survival analysis, and construction of the nomogram respectively. Besides, “pheatmap,” “ggplot2,” and “ggpubr” packages were applied for different plots. *P* < 0.05 was set as statistically significant in most part of our study.

## Results

### Characteristics of GC patients enrolled in this study

Clinical and transcriptome data of 375 GC and 32 normal cases for subsequent analysis were selected and matched by sample ID after they were extracted separately from the TCGA database. The clinical information of 371 matched cases including variables of age, gender, grade, stage, follow-up time, and survival status and the detailed clinicopathologic features were listed in Table 1.

**Table 1:** Clinicopathologic features of patients with GC in this study

Clinicopathologic features	N	%
Age(years)		
≤ 65	163	43.94
> 65	205	55.26
Unknown	3	0.80
Gender		
Male	238	64.15
Female	133	35.85
T classification		
T1	18	4.85
T2	78	21.02
T3	167	45.01
T4	101	27.22
Unknown	7	1.90
N classification		
N0	108	29.11
N1	97	26.15
N2	74	19.95
N3	74	19.95
Unknown	18	4.84
M classification		
M0	328	88.41
M1	25	6.74
Unknown	18	4.85
Histologic grade		
G1	10	2.70
G2	134	36.12
G3	218	58.76
Unknown	9	2.42

### Differentially expressed antioxidant-related genes between GC and normal tissues

According to the four antioxidant-related gene sets from GSEA, gene expressions of all specimens from TCGA database were estimated and 62 antioxidant-related genes were differentially expressed (30 down-regulated and 32 upregulated) in GC tissues (Supplementary Figure 1). Ranked by |logFC|, eight of the top 10 differentially expressed genes were downregulated (APOA4, GSTA3, GSTA2, GSTA1, GSTM5, GPX3, HBA1, and HBB) and the other two genes (APOE and LOXHD1) were upregulated in GC tissues.

### Identification of antioxidant-related prognostic genes in GC patients

Firstly, univariate Cox analysis was utilized to initially select the prognostic genes associated with GC patient overall survival. Four genes, CHAC1 (HR = 0.808, *P* = 0.021), GGT5 (HR = 1.256, *P* = 0.007), GPX8 (HR = 1.349, *P* = 0.002), and PXDN (HR = 1.315, *P* = 0.004), were correlated with GC patients overall survival significantly. Then, further multivariate Cox regression analysis was performed and consequently, two genes CHAC1 (HR = 0.803, *P* < 0.05) and GPX8 (HR = 1.358, *P* < 0.05) were confirmed as independent GC prognostic biomarkers. It can be inferred that CHAC1 acted as a protective role while GPX8 played a risky role.

**Fig. 2:**
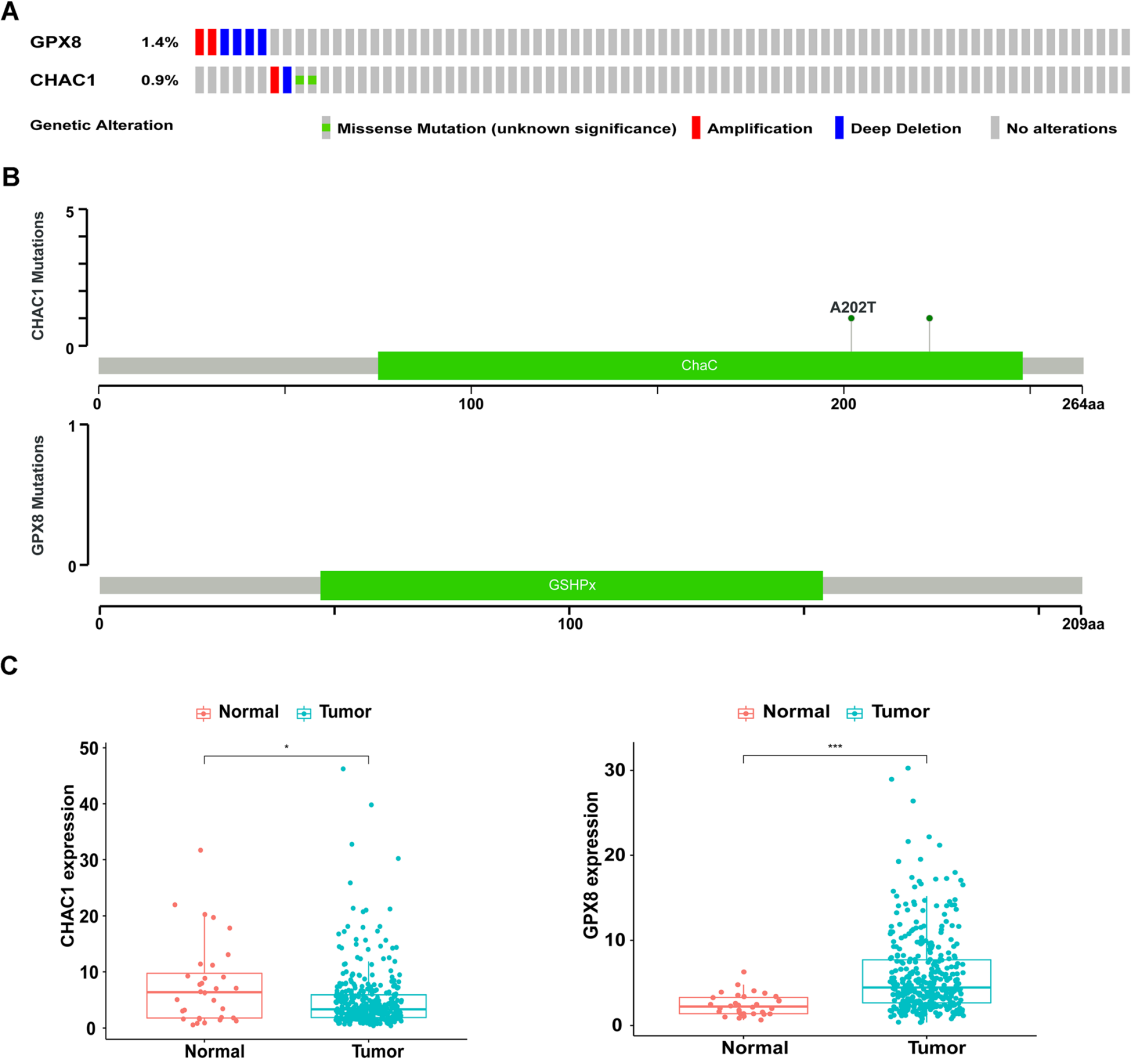
Identification of antioxidant-related genes related to survival of GC patients. **A** Identified genes’ mutation in clinical tissues from TCGA database. **B** Identified genes’ specific mutation sites. **C** Differential expression of the two selected genes (*p < 0.05, ***p < 0.001)

Subsequently, the alternations in the two genes were evaluated by testing the samples from TCGA in cBioPortal database (http://www.cbioprtal.org). The results showed that 10 (2.67%) of all sequenced cases had alternation. Among them, gene GPX8 contained two amplification and four deep deletion alterations. The CHAC1 gene had 1% mutation, including one amplification, one deep deletion, and two missense mutations (Fig. 2A). The specific mutation sites were shown in Fig. 2B. No mutation happens inside the domain of GPX8 gene, but there were two mutation sites inside the domain of CHAC1 gene, which could affect its function.

Besides, the expression of gene CHAC1 and GPX8 between GC and normal tissues were explored. Gene CHAC1 expressed significantly lower in GC compared with normal cases (*P* < 0.05) while gene GPX8 expressed significantly higher in GC cases on the contrary (*P* < 0.01, Fig. 2C). Furthermore, through other databases, we verified the differential expression of the four antioxidant-related genes in GC by Oncomine analysis [20] and their prognostic value using the Kaplan-Meier plotter (www.kmplot.com) [21] (Supplementary Figure 2).

### Construction of antioxidant-related gene signature as a risk model

**Fig. 3:**
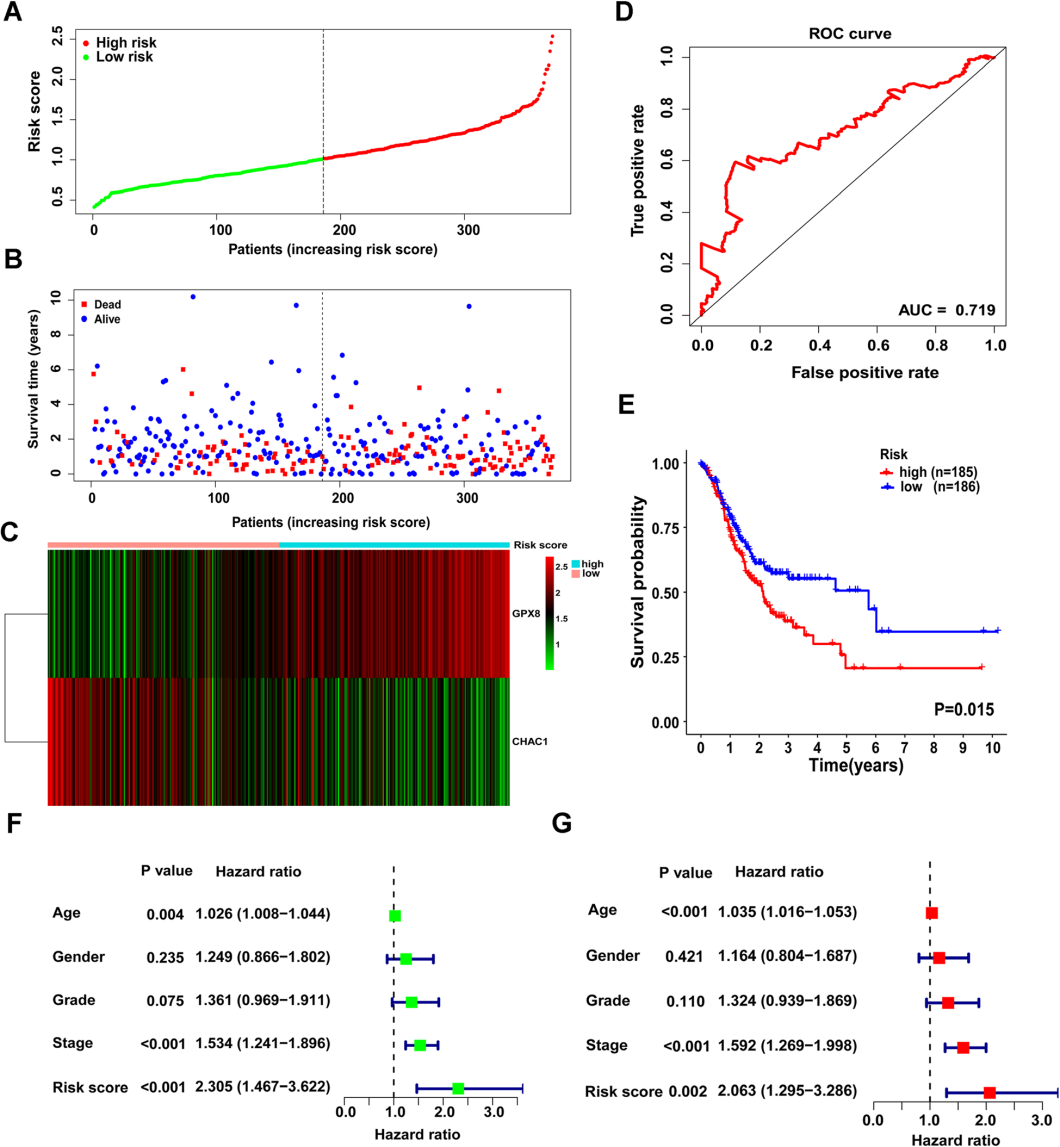
Antioxidant-related gene signature acts as a predictor for GC prognosis. **A** Distribution of risk scores in ascending order of all GC patients: low risk (green) and high risk (red). **B** Relationship between survival time and status. **C** A heatmap of the gene signature’s differential expression profile in two groups. **D** ROC curve analysis to estimate the prognostic efficiency of gene signature. **E** Kaplan-Meier curves of the low- and high-risk group. **F** Univariate regression analysis. **G** Multivariate regression analysis

On the basis of Cox regression analysis, a two-gene signature was established with the risk score which could be calculated as a linear combination of regression coefficient weighted gene expression level of CHAC1 and GPX8: (− 0.2200 × expression of CHAC1) + (0.3058 × expression of GPX8). Risk scores of all the GC patients were calculated and by group median risk score, they were subsequently divided into high- and low-risk teams (Fig. 3A). Distribution of the risk score and survival time was shown in Fig. 3B, and patients in high-risk group showed poorer prognoses than those in low-risk group. In addition, the expression profiles of CHAC1 and GPX8 were shown in a heatmap (Fig. 3C). The expression of the GPX8 gene was upregulated while the expression of the CHAC1 gene was downregulated, along with increasing risk score. Furthermore, a receiver operator characteristic (ROC) curve was drawn which could evaluate the performance of the risk model (Fig. 3D). The area under the curve (AUC) was 0.719, indicating good sensitivity and specificity of the score-based risk model in predicting the prognosis of GC patients. And in overall survival analysis, patients with lower risks were substantiated to have better prognoses by the Kaplan-Meier survival curves and log-rank tests (*P* < 0.05, Fig. 3E).

### Validation of prediction ability of the two-gene signature

Univariate and multivariate Cox analyses then estimated the prognostic value of antioxidant-related gene signature as well as other clinicopathological features of GC patients containing age, gender, grade, and stage. Among these five variables, the results of univariate analysis revealed that age [hazard ratio (HR) = 1.026, 95% confidence interval (CI) 1.008–1.044, *P* = 0.004], stage (HR = 1.534, 95% CI 1.241–1.896, *P* < 0.001) and risk score (HR = 2.305, 95% CI: 1.467-3.622, *P* < 0.001) had significantly close relationship with GC patients prognoses (Fig. 3F). Meanwhile, multivariate analysis revealed that these three features, age (HR = 1.035, 95% CI 1.016–1.053, *P* < 0.001), stage (HR = 1.592, 95% CI 1.269–1.998, *P* < 0.001), and risk score (HR = 2.063, 95% CI 1.295–3.286, *P* = 0.002), were independent prognostic markers (Fig. 3G).

**Fig. 4:**
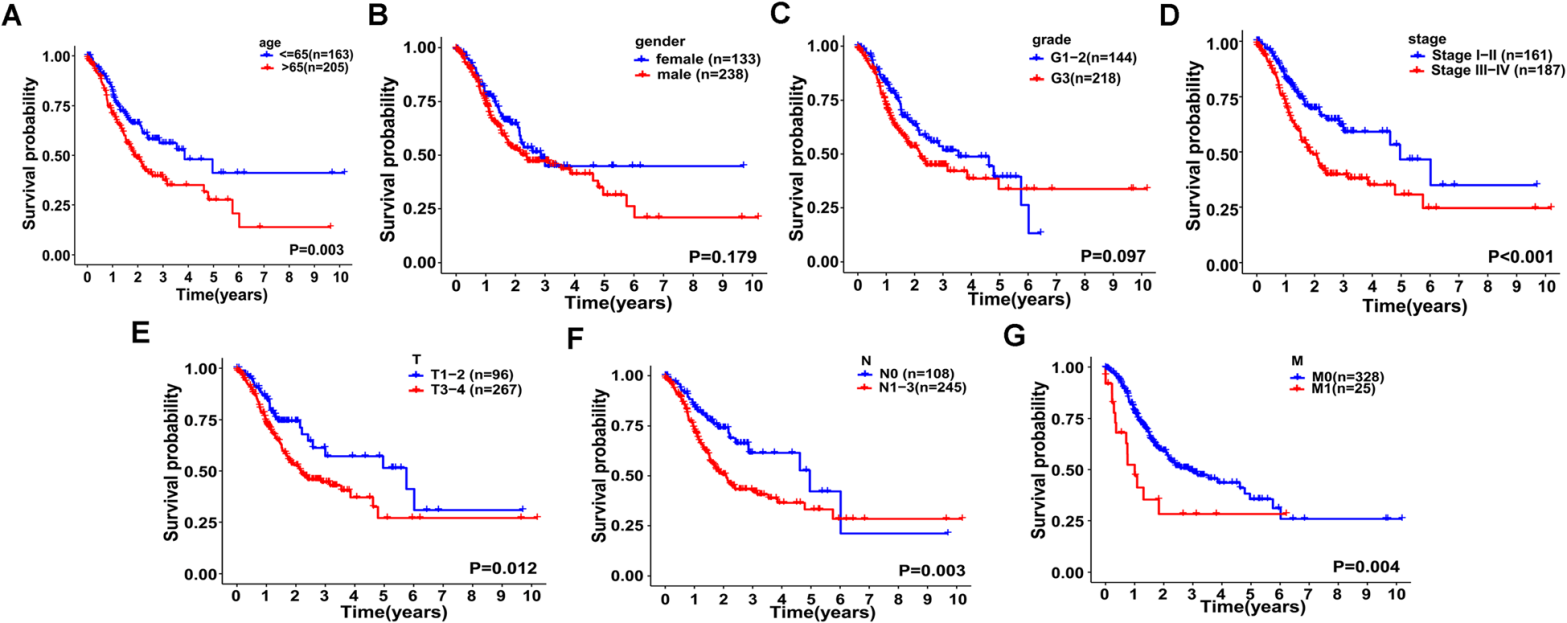
Kaplan-Meier survival analyses in GC subgroups with different clinicopathologic features. **A** Age. **B** Gender. **C** Grade. **D** Stage. **E** T classification. **F** N classification. **G** M classification

According to the previous two regression analyses, age, stage, and risk score were independent predictors for overall survival of GC patients, and these results were further confirmed by Kaplan-Meier survival curves (Fig. 4A–D). Patients > 65 years old and those at III–IV stages manifested a poorer survival probability. And patients at T1-2, N0, and M0 had better prognoses (Fig. 4E–G).

**Fig. 5:**
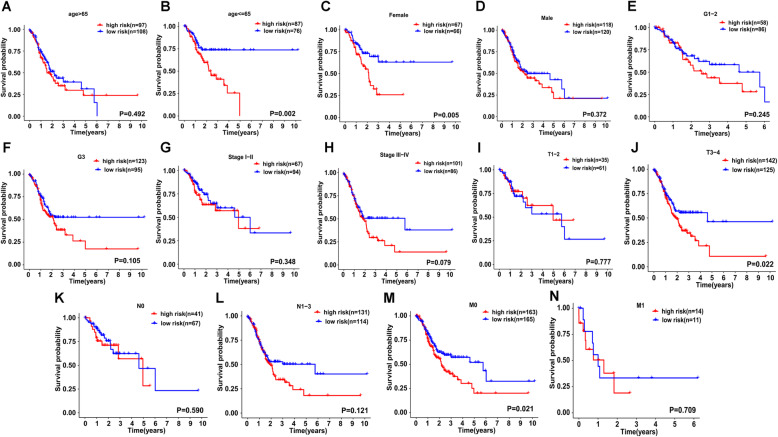
Stratified analyses for prognostic value of the risk model in different GC subgroups. **A** Age > 65. **B** Age ≤ 65. **C** Female. **D** Male. **E** G1-2. **F** G3. **G** Stage I-II. **H** Stage III-IV. **I** T1-2. **J** T3-4. **K** N0. **L** N1-3. **M** M0. **N** M1

Then, further stratified analysis was conducted to confirm the performance of the antioxidant-related gene signature in different subgroups. As shown in the Kaplan-Meier curves (Fig. 5A–N), the two-gene risk model could act as a reliable prognostic predictor for GC patients who were ≤ 65, female, T3-4, and M0 stages by distinguishing patients into high- and low-risk groups.

### Construction and validation of a nomogram model

**Fig. 6:**
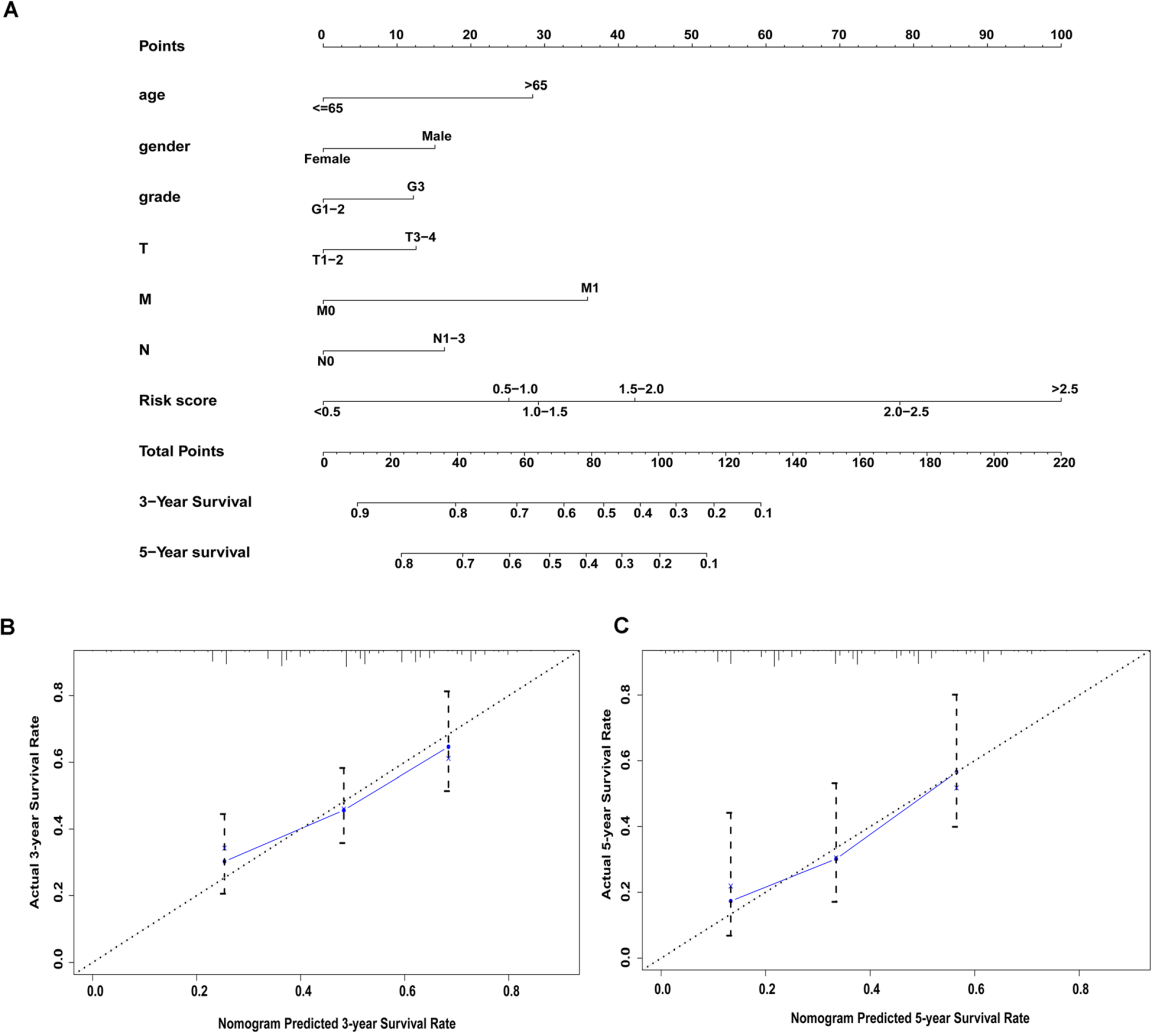
Construction and validation of a nomogram model combing clinicopathologic variables and the antioxidant-related gene signature. **A** The nomogram to predict 3- and 5-year survival probability of GC patients. **B**, **C** The calibration plots to estimate the predictive performance of the nomogram. Nomogram-predicted OS probability is presented on the x-axis; actual survival is presented on the y-axis

A nomogram model for the evaluation of GC patients OS probability was constructed (Fig. 6A), combing clinicopathological features and the antioxidant-related gene signature. Harrell’ concordance index for survival prediction was 0.665 (95% CI 0.614–0.716). And in Fig. 6B, C, the calibration plots verified that both 3- and 5-year OS predictions by nomogram were highly consistent with the actual observation of GC patients. Additionally, we calculated the area under ROC curves of the 3-year (AUC = 0.680) and 5-year (AUC = 0.674) survival prediction to test the specificity and sensitivity of the nomogram model. Besides, to further confirm the consistency and accuracy, we established another test cohort from the TCGA database for internal validation. The nomogram in the testing cohort also showed good prediction performance as the training one and the C-index was 0.706 (95% CI 0.612–0.800). And the area under ROC curves of the 3-year (AUC = 0.703) and 5-year (AUC = 0.641) survival prediction were also calculated.

## Discussion

In normal cells, antioxidant system helps maintain the appropriate level of reactive oxygen species (ROS) through various signaling pathways [22]. But tumor cells are featured with high levels of ROS, which can modulate pathways and change gene epigenetics, influencing various cellular and molecular processes in tumor cells and microenvironment [12]. Antioxidant proteins are also elevated to reach a new redox balance with ROS in tumor cells and maintain a pro-tumorigenic environment [23]. These suggested that antioxidants and ROS are closely related to the beginning and progression of cancer. Increasing researches have focused on the correlation between antioxidant and GC, and some scholars have demonstrated the significant role of antioxidant in GC development. For example, previous research discovered that exogenous antioxidant alpha-lipoic acid (ALA) mediating the expression of MUC4 gene inhibited proliferation and invasion of GC cells [18]. In xenograft models, GC growth can be significantly suppressed after intratumoral injection of an antioxidative enzyme nicotinamide nucleotide transhydrogenase [24]. Furthermore, increasing researches have focused on the antioxidant-related genes in signaling pathways of antioxidant system [25]. The expression of these genes might be crucial to GC development and might enlighten diagnosis, evaluation and treatment of GC, which requires more studies.

In recent years, instead of the traditional predictive methods like TNM stages and pathological grades, scholars showed interest in novel models to assess the prognosis of cancer patient more efficiently and precisely [26]. Recently, molecular biomarkers like mRNAs have been seen as potential prognosis predictors, implying their clinical significance in researches [27, 28]. For instance, expression of MYOZ2 was significantly higher in GC tissues than that in the normal tissues, which might involve in the carcinogenesis of GC [29]. Similarly, excessive level of HBO1 mRNA in GC tissues and its negative correlation with GC patient survival indicated that HBO1 might act as a potential biomarker to predict patient prognosis [30]. Nevertheless, single genes could be affected by multiple factors, and it was insufficient to predict patient prognosis independently based on these individual biomarkers [31, 32]. Therefore, gene signature, a statistical model made up of various marker genes, has been utilized to overcome the limitation of consistency and to predict survival outcome on a combined effect [33]. Some scholars have identified and validated prognostic gene signatures of GC and built up a specific score formula to measure the risk, but these signatures had not been widely accepted or put into practice [34]. And studies on antioxidant-related gene signatures of GC are still absent to date.

Therefore, in the study, we determined two genes (CHAC1 and GPX8) associated with antioxidant system and unraveled their prognostic value in GC by bioinformatics methods. Different from previous predicting tools, this score-based risk model could act as a more efficient indicator for GC patients OS prediction and could help classification and individualized treatment for clinical application. Kaplan-Meier curves verified that patients with higher risks showed worse prognoses. Furthermore, we established a comprehensive nomogram model to provide a more efficient predicting tool in clinical practice and help make a more accurate assessment of GC patients.

As for the two antioxidant-related genes, derived from a family of Cys-glutathione peroxidase, GPX8 mainly resides in mitochondrial endoplasmic reticulum membranes and it supports the folding of oxidative protein [35, 36]. In addition, it can reduce hydrogen peroxide, lipid hydroperoxides, and other damage related to oxidative stress with glutathione (GSH), which was closely associated with carcinogenesis [37, 38]. Scholars discovered the GPX8/IL-6/STAT3 axis as an essential pathway in regulating cell aggressiveness of breast cancer [39]. And in GC, expression of GPX8 has been proved to increase in GC patients with worse OS, and it was confirmed to be an independent prognosis predictor [40], in accord with our result. However, its regulatory pathway and cellular functions have not been fully elucidated. CHAC1, a newly discovered enzyme associated with γ-glutamyl cyclotransferase activity, could degrade intracellular GSH, which might cause oxidative stress and contribute to necroptosis and ferroptosis in cancer [41, 42]. In previous studies, higher expression of CHAC1 could act as a protective role in accelerating apoptotic death of glioma through various pathways [43], and it was suggested to be included in prognostic prediction to aid the scheme of treatment in breast cancer [44]. Our results also indicated the protective role of CHAC1 and showed significant predictive value. Contradictorily, some scholars found the overexpression of CHAC1 in *H. pylori*-infected parietal cells could increase the risk of GC [45], but overall, there are few studies and direct evidence illustrating the relationship between CHAC1 and GC. As analyzed above, these two key antioxidant-related enzymes act as important parts in the growth and proliferation of GC and show prognostic value in GC patients. Furthermore, oxidative stress and antioxidant system play a vital part in the tumorigenesis and progression of GC.

In conclusions, an antioxidant-related gene signature was firstly identified, and GC patient prognoses could be quantified by this risk model more efficiently and accurately. Nomogram integrating the gene signature with clinical factors provides an efficient tool in predicting prognosis of GC patients in clinical practice. Our results help enlighten the potential mechanisms of molecular antioxidant system in GC progression and offer more potential biomarkers for early diagnostic and therapeutic targets for GC treatment.

## Supplementary Information

**Additional file 1: Supplementary Figure 1.** The heatmap of expression profiles for the differentially expressed antioxidant-related genes between GC and normal cases.

**Additional file 2: Supplementary Figure 2.** Validation of the differential expression and prognostic value of the four antioxidant-related genes in GC. The differential expression of gene (A) CHAC1, (B) GGT5, (C) GPX8 and (D) PXDN between gastric tissue and GC were analyzed by Oncomine. The survival analysis of GC patients with high or low expression of gene (E) CHAC1, (F) GGT5, (G) GPX8 and (H) PXDN was conducted by Kaplan-Meier plotter.

**Additional file 3: Supplementary Data 1.** Original data of clinical information.

**Additional file 4: Supplementary Data 2.** Original data of gene expression.

**Additional file 5: Supplementary Data 3.** R language code.

## Data Availability

The information of this study here is obtained from the TCGA (https://portal.gdc.cancer.gov/) and cBioportal (http://www.cbioportal.org/).

## Abbreviations

GC: Gastric cancer
TCGA: The Cancer Genome Atlas
GSEA: Gene set enrichment analysis
ROS: Reactive oxygen species
AUC: Areas under the curve
ROC: Receiver operating characteristic
HR: Hazard ratio
C-index: Concordance index
CI: Confidence interval
OS: Overall survival
