# Supplementary figures and images for: Identification of novel antioxidant gene signature to predict the prognosis of patients with gastric cancer
Source: World J Surg Oncol. 2021 Jul 20;19:219. doi: 10.1186/s12957-021-02328-w (PMC8293592; doi:10.1186/s12957-021-02328-w)

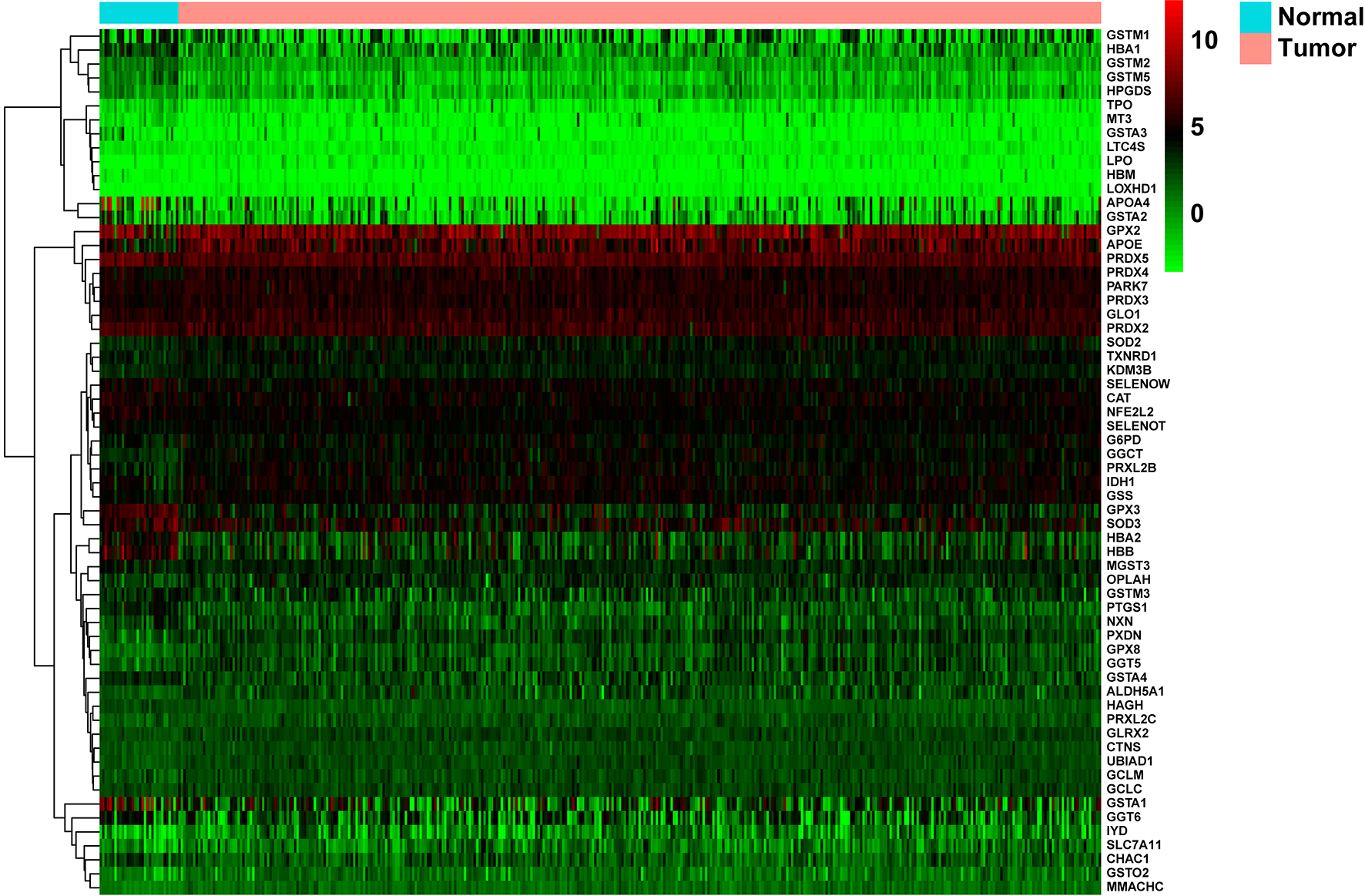

Supplement: Supplementary file 1 — Additional file 1: Supplementary Figure 1. The heatmap of expression profiles for the differentially expressed antioxidant-related genes between GC and normal cases. [file 12957_2021_2328_MOESM1_ESM.tif]

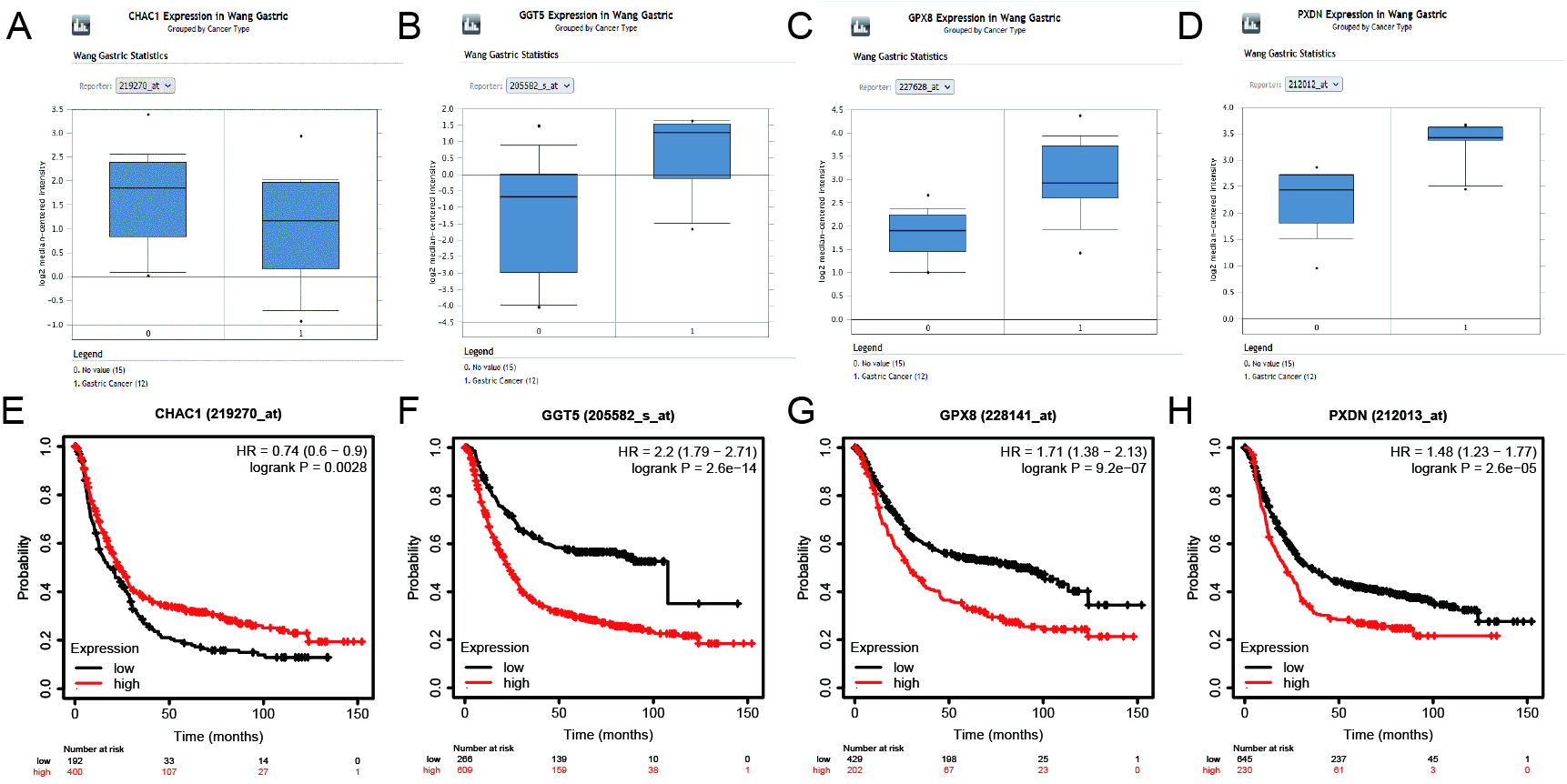

Supplement: Supplementary file 2 — Additional file 2: Supplementary Figure 2. Validation of the differential expression and prognostic value of the four antioxidant-related genes in GC. The differential expression of gene (A) CHAC1, (B) GGT5, (C) GPX8 and (D) PXDN between gastric tissue and GC were analyzed by Oncomine. The survival analysis of GC patients with high or low expression of gene (E) CHAC1, (F) GGT5, (G) GPX8 and (H) PXDN was conducted by Kaplan-Meier plotter. [file 12957_2021_2328_MOESM2_ESM.tif]
