# Supplementary material for: Identification of novel antioxidant gene signature to predict the prognosis of patients with gastric cancer
Source: World J Surg Oncol. 2021 Jul 20;19:219. doi: 10.1186/s12957-021-02328-w (PMC8293592; doi:10.1186/s12957-021-02328-w)
Supplement: Supplementary file 5 — Additional file 5: Supplementary Data 3. R language code. [file 12957_2021_2328_MOESM5_ESM.docx]

R language code

1. Differential analysis of antioxidant-related genes

library("limma")

setwd("D:\\GC\\process\\diff")

inputFile="GeneExp.txt"

pvalFilter=0.05

logFCfilter=0

conNum=32

treatNum=375

outTab=data.frame()

grade=c(rep(1,conNum),rep(2,treatNum))

rt=read.table(inputFile,sep="\t",header=T,check.names=F)

rt=as.matrix(rt)

rownames(rt)=rt[,1]

exp=rt[,2:ncol(rt)]

dimnames=list(rownames(exp),colnames(exp))

data=matrix(as.numeric(as.matrix(exp)),nrow=nrow(exp),dimnames=dimnames)

data=avereps(data)

data=data[rowMeans(data)>0,]

for(i in row.names(data)){

geneName=unlist(strsplit(i,"\\|",))[1]

geneName=gsub("\\/", "_", geneName)

rt=rbind(expression=data[i,],grade=grade)

rt=as.matrix(t(rt))

wilcoxTest<-wilcox.test(expression ~ grade, data=rt)

conGeneMeans=mean(data[i,1:conNum])

treatGeneMeans=mean(data[i,(conNum+1):ncol(data)])

logFC=log2(treatGeneMeans)-log2(conGeneMeans)

pvalue=wilcoxTest$p.value

conMed=median(data[i,1:conNum])

treatMed=median(data[i,(conNum+1):ncol(data)])

diffMed=treatMed-conMed

if( ((logFC>0) & (diffMed>0)) | ((logFC<0) & (diffMed<0)) ){

outTab=rbind(outTab,cbind(gene=i,conMean=conGeneMeans,treatMean=treatGeneMeans,logFC=logFC,pValue=pvalue))

}

}

write.table(outTab,file="all.txt",sep="\t",row.names=F,quote=F)

outDiff=outTab[( abs(as.numeric(as.vector(outTab$logFC)))>logFCfilter & as.numeric(as.vector(outTab$pValue))<pvalFilter),]

write.table(outDiff,file="diff.xls",sep="\t",row.names=F,quote=F)

write.table(outDiff,file="diff.txt",sep="\t",row.names=F,quote=F)

heatmap=rbind(ID=colnames(data[as.vector(outDiff[,1]),]),data[as.vector(outDiff[,1]),])

write.table(heatmap,file="diffGeneExp.txt",sep="\t",col.names=F,quote=F)

2. Merge the differential expressed genes and the clinical information

library(limma)

expFile="diffGeneExp.txt"

cliFile="time.txt"

setwd("D:\\GC\\process\\ mergeTime")

rt=read.table(expFile,sep="\t",header=T,check.names=F)

rt=as.matrix(rt)

rownames(rt)=rt[,1]

exp=rt[,2:ncol(rt)]

dimnames=list(rownames(exp),colnames(exp))

data=matrix(as.numeric(as.matrix(exp)),nrow=nrow(exp),dimnames=dimnames)

data=avereps(data)

data=data[rowMeans(data)>0,]

group=sapply(strsplit(colnames(data),"\\-"),"[",4)

group=sapply(strsplit(group,""),"[",1)

group=gsub("2","1",group)

data=data[,group==0]

colnames(data)=gsub("(.*?)\\-(.*?)\\-(.*?)\\-(.*?)\\-.*","\\1\\-\\2\\-\\3",colnames(data))

data=t(data)

data=avereps(data)

cli=read.table(cliFile,sep="\t",check.names=F,header=T,row.names=1)

sameSample=intersect(row.names(data),row.names(cli))

data=data[sameSample,]

cli=cli[sameSample,]

out=cbind(cli,risk)

out=cbind(id=row.names(out),out)

write.table(out,file="expTime.txt",sep="\t",row.names=F,quote=F)

3. Univariate Cox regression analysis

library(survival)

setwd("D:\\GC\\process\\uniCox")

pFilter=0.05

rt=read.table("expTime.txt",header=T,sep="\t",check.names=F,row.names=1) rt$futime=rt$futime/365

rt[,3:ncol(rt)]=log2(rt[,3:ncol(rt)]+1)

diffRT=read.table("diff.txt",header=T,sep="\t",check.names=F,row.names=1)

outTab=data.frame()

sigGenes=c("futime","fustat")

for(gene in colnames(rt[,3:ncol(rt)])){

if(sd(rt[,gene])<0.01){next}

cox=coxph(Surv(futime, fustat) ~ rt[,gene], data = rt)

coxSummary = summary(cox)

coxP=coxSummary$coefficients[,"Pr(>|z|)"]

if( ((coxSummary$conf.int[,"exp(coef)"]>1) & (diffRT[gene,"logFC"]>0)) | ((coxSummary$conf.int[,"exp(coef)"]<1) & (diffRT[gene,"logFC"]<0)) ){

if(coxP<pFilter){

group=ifelse(rt[,gene]>median(rt[,gene]),"high","low")

diff=survdiff(Surv(futime, fustat) ~group,data = rt)

pValue=1-pchisq(diff$chisq,df=1)

if(pValue<pFilter){

sigGenes=c(sigGenes,gene)

outTab=rbind(outTab,

cbind(gene=gene,

HR=coxSummary$conf.int[,"exp(coef)"],

HR.95L=coxSummary$conf.int[,"lower .95"],

HR.95H=coxSummary$conf.int[,"upper .95"],

coxPvalue=coxP) )

}

}

}

}

write.table(outTab,file="uniCox.txt",sep="\t",row.names=F,quote=F) surSigExp=rt[,sigGenes]

surSigExp=cbind(id=row.names(surSigExp),surSigExp)

write.table(surSigExp,file="uniSigExp.txt",sep="\t",row.names=F,quote=F)

4. Multivariate Cox regression analysis

library(survival)

setwd("D:\\GC\\process\\multiCox")

rt=read.table("uniSigExp.txt",header=T,sep="\t",check.names=F,row.names=1)

multiCox=coxph(Surv(futime, fustat) ~ ., data = rt)

multiCox=step(multiCox,direction = "both")

multiCoxSum=summary(multiCox)

outTab=data.frame()

outTab=cbind(

coef=multiCoxSum$coefficients[,"coef"],

HR=multiCoxSum$conf.int[,"exp(coef)"])

outTab=cbind(id=row.names(outTab),outTab)

outTab=gsub("`","",outTab)

write.table(outTab,file="multiCox.xls",sep="\t",row.names=F,quote=F)

riskScore=predict(multiCox,type="risk",newdata=rt)

coxGene=rownames(multiCoxSum$coefficients)

coxGene=gsub("`","",coxGene)

outCol=c("futime","fustat",coxGene)

risk=as.vector(ifelse(riskScore>median(riskScore),"high","low"))

write.table(cbind(id=rownames(cbind(rt[,outCol],riskScore,risk)),cbind(rt[,outCol],riskScore,risk)),

file="risk.txt",

sep="\t",

quote=F,

row.names=F)

5.Survival analysis of the signature

library(survival)

library(survminer)

setwd("D:\\GC\\process\\survival")

rt=read.table("risk.txt",header=T,sep="\t")

diff=survdiff(Surv(futime, fustat) ~risk,data = rt)

pValue=1-pchisq(diff$chisq,df=1)

if(pValue<0.001){

pValue="p<0.001"

}else{

pValue=paste0("p=",sprintf("%.03f",pValue))

}

tab=table(rt$risk)

labels=paste0(names(tab),"(n=",tab,")")

fit <- survfit(Surv(futime, fustat) ~ risk, data = rt)

pdf(file="survival.pdf",onefile = FALSE,

width = 5.5,

height =5)

ggsurvplot(fit,

data=rt,

pval=pValue,

pval.size=6,

legend.labs=labels,

legend.title="Risk",

xlab="Time(years)",

break.time.by = 1,

palette=c("red", "blue") )

dev.off()

6.ROC curve analysis

library(survivalROC)

setwd("D:\\GC\\process\\ROC")

rt=read.table("riskscore.txt",header=T,sep="\t",check.names=F,row.names=1)

pdf(file="ROC.pdf",width=6,height=6)

par(oma=c(0.5,1,0,1),font.lab=1.5,font.axis=1.5)

roc=survivalROC(Stime=rt$futime, status=rt$fustat, marker = rt$risk_score,

predict.time =5, method="KM")

plot(roc$FP, roc$TP, type="l", xlim=c(0,1), ylim=c(0,1),col='red',

xlab="False positive rate", ylab="True positive rate",

main=paste("ROC curve (", "AUC = ",sprintf("%.3f",roc$AUC),")"),

lwd = 2, cex.main=1.3, cex.lab=1.2, cex.axis=1.2, font=1.2)

abline(0,1)

dev.off()

7.Draw the risk plots

library(pheatmap)

setwd("D:\\GC\\process\\riskPlot")

rt=read.table("risk.txt",sep="\t",header=T,row.names=1,check.names=F)

rt=rt[order(rt$riskScore),]

riskClass=rt[,"risk"]

lowLength=length(riskClass[riskClass=="low"])

highLength=length(riskClass[riskClass=="high"])

line=rt[,"riskScore"]

line[line>10]=10

pdf(file="riskScore.pdf",width = 10,height = 5)

plot(line,

type="p",

pch=20,

xlab="Patients (increasing risk socre)",

ylab="Risk score",

col=c(rep("green",lowLength),

rep("red",highLength)))

abline(v=lowLength,lty=5)

legend("topleft", c("High risk", "low Risk"),bty="n",pch=19,col=c("red","green"),cex=1.2)

dev.off()

color=as.vector(rt$fustat)

color[color==1]="red"

color[color==0]="blue"

pch=as.vector(rt$fustat)

pch[pch==1]=15

pch[pch==0]=19

pdf(file="survStat.pdf",width = 10,height = 5)

plot(rt$futime,

pch=pch,

xlab="Patients (increasing risk socre)",

ylab="Survival time (years)",

col=color)

legend("topleft", c("Dead", "Alive"),bty="n",pch=c(15,19),col=c("red","blue"),cex=1.2)

abline(v=lowLength,lty=2)

dev.off()

rt1=rt[c(3:(ncol(rt)-2))]

rt1=t(rt1)

rt1=log2(rt1+1)

annotation=data.frame(type=rt[,ncol(rt)])

rownames(annotation)=rownames(rt)

pdf(file="heatmap.pdf",width = 10,height = 5)

pheatmap(rt1,

annotation=annotation,

cluster_cols = FALSE,

fontsize_row=11,

show_colnames = F,

fontsize=7,

fontsize_col=3,

color = colorRampPalette(c("green", "black", "red"))(50) )

dev.off()

8.Draw the forest plots

library(survival)

setwd("D:\\GC\\process\\indep")

risk=read.table("risk.txt",header=T,sep="\t",check.names=F,row.names=1)

cli=read.table("clinical.txt",sep="\t",check.names=F,header=T,row.names=1)

sameSample=intersect(row.names(cli),row.names(risk))

risk=risk[sameSample,]

cli=cli[sameSample,]

rt=cbind(futime=risk[,1],fustat=risk[,2],cli,riskScore=risk[,(ncol(risk)-1)])

uniTab=data.frame()

for(i in colnames(rt[,3:ncol(rt)])){

cox <- coxph(Surv(futime, fustat) ~ rt[,i], data = rt)

coxSummary = summary(cox)

uniTab=rbind(uniTab,

cbind(id=i,

HR=coxSummary$conf.int[,"exp(coef)"],

HR.95L=coxSummary$conf.int[,"lower .95"],

HR.95H=coxSummary$conf.int[,"upper .95"],

pvalue=coxSummary$coefficients[,"Pr(>|z|)"])

)

}

write.table(uniTab,file="uniCox.txt",sep="\t",row.names=F,quote=F)

multiCox=coxph(Surv(futime, fustat) ~ ., data = rt)

multiCoxSum=summary(multiCox)

multiTab=data.frame()

multiTab=cbind(

HR=multiCoxSum$conf.int[,"exp(coef)"],

HR.95L=multiCoxSum$conf.int[,"lower .95"],

HR.95H=multiCoxSum$conf.int[,"upper .95"],

pvalue=multiCoxSum$coefficients[,"Pr(>|z|)"])

multiTab=cbind(id=row.names(multiTab),multiTab)

write.table(multiTab,file="multiCox.txt",sep="\t",row.names=F,quote=F)

bioForest=function(coxFile=null,forestFile=null,forestCol=null){

rt <- read.table(coxFile,header=T,sep="\t",row.names=1,check.names=F)

gene <- rownames(rt)

hr <- sprintf("%.3f",rt$"HR")

hrLow <- sprintf("%.3f",rt$"HR.95L")

hrHigh <- sprintf("%.3f",rt$"HR.95H")

Hazard.ratio <- paste0(hr,"(",hrLow,"-",hrHigh,")")

pVal <- ifelse(rt$pvalue<0.001, "<0.001", sprintf("%.3f", rt$pvalue))

pdf(file=forestFile, width = 6,height = 4.3)

n <- nrow(rt)

nRow <- n+1

ylim <- c(1,nRow)

layout(matrix(c(1,2),nc=2),width=c(3,2.5))

xlim = c(0,3)

par(mar=c(4,2,2,1))

plot(1,xlim=xlim,ylim=ylim,type="n",axes=F,xlab="",ylab="")

text.cex=0.8

text(0,n:1,gene,adj=0,cex=text.cex)

text(1.5-0.5*0.2,n:1,pVal,adj=1,cex=text.cex);text(1.5-0.5*0.2,n+1,'pvalue',cex=text.cex,font=2,adj=1)

text(3,n:1,Hazard.ratio,adj=1,cex=text.cex);text(3,n+1,'Hazard ratio',cex=text.cex,font=2,adj=1,)

par(mar=c(4,1,2,1),mgp=c(2,0.5,0))

xlim = c(0,max(as.numeric(hrLow),as.numeric(hrHigh)))

plot(1,xlim=xlim,ylim=ylim,type="n",axes=F,ylab="",xaxs="i",xlab="Hazard ratio")

arrows(as.numeric(hrLow),n:1,as.numeric(hrHigh),n:1,angle=90,code=3,length=0.05,col="darkblue",lwd=3.5)

abline(v=1,col="black",lty=2,lwd=2)

boxcolor = ifelse(as.numeric(hr) > 1, forestCol, forestCol)

points(as.numeric(hr), n:1, pch = 15, col = boxcolor, cex=2)

axis(1)

dev.off()

}

bioForest(coxFile="uniCox.txt",forestFile="uniForest.pdf",forestCol="green")

bioForest(coxFile="multiCox.txt",forestFile="multiForest.pdf",forestCol="red")

9. Scatter plots of the expression of the signature genes

library(limma)

library(ggpubr)

setwd("D:\\GC\\process\\scatter")

expFile="GeneExp.txt"

riskFile="risk.txt"

conNum=32

treatNum=375

outTab=data.frame()

Group=c(rep("Normal",conNum),rep("Tumor",treatNum))

rt=read.table(expFile,sep="\t",header=T,check.names=F)

rt=as.matrix(rt)

rownames(rt)=rt[,1]

exp=rt[,2:ncol(rt)]

dimnames=list(rownames(exp),colnames(exp))

data=matrix(as.numeric(as.matrix(exp)),nrow=nrow(exp),dimnames=dimnames)

data=avereps(data)

data=data[rowMeans(data)>0,]

risk=read.table(riskFile,sep="\t",header=T,row.names=1,check.names=F)

modelGene=colnames(risk)[3:(ncol(risk)-2)]

data=t(data[modelGene,])

data=cbind(data,Group)

data=rbind(ID=colnames(data),data)

write.table(data,file="data.txt",sep="\t",col.names=F,quote=F)

data=read.table("data.txt",sep="\t",header=T,row.names=1,check.names=F)

for(gene in colnames(data)[1:(ncol(data)-1)]){

subData=data[,c(gene,"Group")]

colnames(subData)=c("gene","Group")

group=levels(factor(subData$Group))

comp=combn(group,2)

my_comparisons=list()

for(i in 1:ncol(comp)){my_comparisons[[i]]<-comp[,i]}

boxplot=ggboxplot(subData, x="Group", y="gene", color="Group",

xlab="",

ylab=paste(gene,"expression"),

legend.title="",

add = "jitter")+

stat_compare_means(comparisons = my_comparisons,symnum.args=list(cutpoints = c(0, 0.001, 0.01, 0.05, 1), symbols = c("***", "**", "*", "ns")),label = "p.signif")

pdf(file=paste0(gene,".pdf"),width=5.5,height=5)

print(boxplot)

dev.off()

}

10.Survival analysis of clinical characteristics

library(survival)

library(survminer)

setwd("D:\\GC\\process\\cliSurvival")

risk=read.table("risk.txt",header=T,sep="\t",check.names=F,row.names=1)

cli=read.table("clinical.txt",sep="\t",check.names=F,header=T,row.names=1)

sameSample=intersect(row.names(cli),row.names(risk))

risk=risk[sameSample,]

cli=cli[sameSample,]

data=cbind(futime=risk[,1],fustat=risk[,2],cli)

for(i in colnames(data[,3:ncol(data)])){

rt=data[,c("futime","fustat",i)]

rt=rt[(rt[,i]!="unknow"),]

colnames(rt)=c("futime","fustat","clinical")

tab=table(rt[,"clinical"])

tab=tab[tab!=0]

labels=paste0(names(tab),"(n=",tab,")")

diff=survdiff(Surv(futime, fustat) ~clinical,data = rt)

pValue=1-pchisq(diff$chisq,df=1)

if(pValue<0.001){

pValue="p<0.001"

}else{

pValue=paste0("p=",sprintf("%.03f",pValue))

}

fit <- survfit(Surv(futime, fustat) ~ clinical, data = rt)

surPlot=ggsurvplot(fit,

data=rt,

pval=pValue,

pval.size=6,

legend.labs=labels,

legend.title=i,

xlab="Time(years)",

break.time.by = 1,

palette=c("blue","red") )

pdf(file=paste0("survival.",i,".pdf"),onefile = FALSE,

width = 5,

height =4.5)

print(surPlot)

dev.off()

}

11.Stratified survival analysis

library(survival)

library(survminer)

setwd("D:\\GC\\process\\cliGroupTest")

risk=read.table("risk.txt",header=T,sep="\t",check.names=F,row.names=1)

cli=read.table("clinical.txt",sep="\t",check.names=F,header=T,row.names=1)

sameSample=intersect(row.names(cli),row.names(risk))

risk=risk[sameSample,]

cli=cli[sameSample,]

data=cbind(futime=risk[,1],fustat=risk[,2],cli,risk=risk[,"risk"])

for(i in colnames(data[,3:(ncol(data)-1)])){

rt=data[,c("futime","fustat",i,"risk")]

rt=rt[(rt[,i]!="unknow"),]

colnames(rt)=c("futime","fustat","clinical","risk")

tab=table(rt[,"clinical"])

tab=tab[tab!=0]

for(j in names(tab)){

rt1=rt[(rt[,"clinical"]==j),]

tab1=table(rt1[,"risk"])

tab1=tab1[tab1!=0]

labels=paste0(names(tab1)," risk(n=",tab1,")")

if(length(labels)==2){

titleName=j

if((i=="age") | (i=="Age") | (i=="AGE")){

titleName=paste0("age",j)

}

diff=survdiff(Surv(futime, fustat) ~risk,data = rt1)

pValue=1-pchisq(diff$chisq,df=1)

if(pValue<0.001){

pValue="p<0.001"

}else{

pValue=paste0("p=",sprintf("%.03f",pValue))

}

fit <- survfit(Surv(futime, fustat) ~ risk, data = rt1)

surPlot=ggsurvplot(fit,

data=rt1,

pval=pValue,

pval.size=6,

legend.labs=labels,

legend.title=titleName,

font.legend=13,

xlab="Time(years)",

break.time.by = 1,

palette=c("red","blue") )

j=gsub(">=","ge",j);j=gsub("<=","le",j);j=gsub(">","gt",j);j=gsub("<","lt",j)

pdf(file=paste0("survival.",i,"_",j,".pdf"),onefile = FALSE,

width = 5,

height =4.5)

print(surPlot)

dev.off()

}

}

}

12.Construction of the nomogram

setwd("D:\\GC\\process\\Nomogram")

library(rms)

library(foreign)

library(survival)

tcga<-read.table("clinical.txt",header=T,sep="\t")

tcga$age<-factor(tcga$age,labels=c("<=65",">65"))

tcga$gender<-factor(tcga$gender,labels=c("FEMALE","MALE"))

tcga$grade<-factor(tcga$grade,labels=c("G1-2","G3"))

tcga$T<-factor(tcga$T,labels=c("T1-2","T3-4"))

tcga$M<-factor(tcga$M,labels=c("M0","M1"))

tcga$N<-factor(tcga$N,labels=c("N0","N1-3"))

tcga$riskScore<-factor(tcga$riskScore,labels=c("<0.5","0.5-1.0","1.0-1.5","1.5-2.0","2.0-2.5",">2.5"))

ddist <- datadist(tcga)

options(datadist='ddist')

cox <- cph(Surv(survival_time,status) ~age + gender + grade + T + M + N + riskScore,surv=T,x=T, y=T,data=tcga)

surv <- Survival(cox)

surv <- Survival(cox)

sur_3_year<-function(x)surv(1*365*3,lp=x)

sur_5_year<-function(x)surv(1*365*5,lp=x)

nom_sur <- nomogram(cox,fun=list(sur_3_year,sur_5_year),lp= F,funlabel=c('3-Year Survival','5-Year survival'),maxscale=100,fun.at=c('0.9','0.8','0.7','0.6','0.5','0.4','0.3','0.2','0.1'))

pdf("nom.pdf",15,10)

plot(nom_sur,xfrac=0.25)

dev.off()

13.Validation of the nomogram

library(foreign)

library(survival)

library(caret)

library(rms)

setwd("D:\\GC\\process\\verification")

tcga<-read.table("clinical.txt",header=T,sep="\t")

set.seed(131)

tcgad<-createDataPartition(y=tcga$id,p=0.70,list=F)

tcgadev<-tcga[tcgad, ]

tcgav<-tcga[-tcgad,]

write.csv(tcgadev, "tcgadev.csv")

write.csv(tcgav, "tcgav.csv")

tcga<-read.table("av.txt",header=T,sep="\t")

tcga$age<-factor(tcga$age,labels=c("<=65",">65"))

tcga$gender<-factor(tcga$gender,labels=c("FEMALE","MALE"))

tcga$grade<-factor(tcga$grade,labels=c("G1-2","G3"))

tcga$T<-factor(tcga$T,labels=c("T1-2","T3-4"))

tcga$M<-factor(tcga$M,labels=c("M0","M1"))

tcga$N<-factor(tcga$N,labels=c("N0","N1-3"))

tcga$riskScore<-factor(tcga$riskScore,labels=c("<0.5","0.5-1.0","1.0-1.5","1.5-2.0","2.0-2.5",">2.5"))

ddist <- datadist(tcga)

options(datadist='ddist')

cox <- cph(Surv(survival_time,status) ~age + gender + grade + T + M + N + riskScore,surv=T,x=T, y=T,data=tcga)

surv <- Survival(cox)

surv <- Survival(cox)

sur_3_year<-function(x)surv(1*365*3,lp=x)

sur_5_year<-function(x)surv(1*365*5,lp=x)

nom_sur <- nomogram(cox,fun=list(sur_3_year,sur_5_year),lp= F,funlabel=c('3-Year Survival','5-Year survival'),maxscale=100,fun.at=c('0.9','0.8','0.7','0.6','0.5','0.4','0.3','0.2','0.1'))

pdf("nom.pdf",15,10)

plot(nom_sur,xfrac=0.25)

dev.off()
